# Enhancement of lipid stability of broiler breast meat and meat products fed on alpha lipoic acid and alpha tocopherol acetate supplemented feed

**DOI:** 10.1186/1476-511X-11-57

**Published:** 2012-05-28

**Authors:** Muhammad Sohaib, Faqir Muhammad Anjum, Muhammad Issa Khan, Muhammad Sajid Arshad, Muhammad Shahid

**Affiliations:** 1 National Institute of Food Science and Technology, University of Agriculture, Faisalabad, Pakistan; 2 Department of Chemistry and Biochemistry, University of Agriculture, Faisalabad, Pakistan

**Keywords:** Lipoic acid, Alpha tocopherol acetate, Breast meat, Nuggets, Patties

## Abstract

This study was designed to investigate the effect of alpha lipoic acid (ALA) and alpha tocopherol acetate (ATA) on the antioxidant potential, lipid stability and the quality of the broiler breast meat and meat products. The treatment plan was as (T_1_ = control feed, T_2_ = 200 mg ATA + 25 mg ALA/kg feed, T_3_ = 200 mg ATA + 75 mg ALA/kg feed, T_4_ = 200 mg ATA + 150 mg ALA/kg feed, T_5_ = Oxidized oil (4%), T_6_ = 200 mg ATA + 150 mg ALA + Oxidized oil (4%)/kg feed). After two weeks of acclimatization the birds were fed with ALA and ATA enriched diet. The results revealed that maximum deposition of ALA took place in T_4_ which contain maximum dose of ALA. The TBARS and DPPH values of the broiler breast meat were in T_4_ (0.14 ± 0.01 MDA/kg of meat, 76.69 ± 0.14%) and in T_5_ were (0.24 ± 0.15 MDA/Kg of meat, 44.98 ± 0.04%) accordingly. ATA concentration were also highest in T_4_ (206.43 ± 0.22 mg/g of meat) and lowest in T_5_ (79.09 ± 0.06 mg/g of meat). Sensory evaluation results showed that nuggets and patties made of T_5_ containing oxidized oil were least liked and T_4_ got highest score. In a nutshell, 150 mg/kg feed dietary supplementation of ALA with constant level of ATA can ameliorate the antioxidant potential, lipid stability and nutritional qualities of broiler breast meat and meat products.

## Introduction

Lipid peroxidation is one of the pivotal mechanisms of quality deterioration in stored foods. The changes in quality resulted from lipid oxidation are deterioration in flavor, color, texture, and nutritive value and the production of toxic compounds [1]. The oxidative stability of meat and meat products depends upon the balance of anti- and pro-oxidants and the oxidation substrates including polyunsaturated fatty acids (PUFA), cholesterol, proteins and pigments [2]. Antioxidants can be used to reduce lipid peroxidation in meat but the use of cocktails of antioxidants may have superior effects as compared to single antioxidants because two or more antioxidants together can act synergistically [3]. Alpha-lipoic acid (ALA) is a coenzyme involved in mitochondrial metabolism. The reduced form of ALA, dihydrolipoic acid, is a powerful mitochondrial antioxidant [4]. ALA is considered an important antioxidant that may be of therapeutic value in some pathologic conditions related to the overproduction of oxidant radicals [5]. ALA is an essential cofactor for mitochondrial enzymes and also a natural antioxidant that is used to quench free radicals [6]. ALA has valuable potential in clinical interests as a cellular thiol-replenishing and redox-modulating agent [7]. The reduced form of ALA, dihydrolipoic acid, reacts with oxidants such as superoxide radicals, hydroxyl radicals, hypochlorous acid, peroxyl radicals, and singlet oxygen [8]. Alpha-tocopherol acetate (ATA) is the most efficient chain-breaking, fat-soluble antioxidant in the tissues [9,10]. Unlike other fat-soluble vitamins, α-tocopherol is not accumulated in the body to toxic levels. Importantly, the body has the ability to eliminate “excess” α-tocopherol by increased metabolism and excretion to prevent excess accumulation of both α-tocopherol and 2,5,7,8-tetramethyl-2-(2′-carboxyethyl)-6-hydroxychroman (α-CEHC) the final product of ATA metabolism, even when daily pharmacologic vitamin E doses are administered [11]. Alpha-lipoic acid and vitamin E have shown synergistic effects against lipid peroxidation by oxidant radicals in several pathological conditions such as a thromboembolic stroke model in rats for neurological functions, glial reactivity and neuronal remodeling [12]. In the present study, broiler birds were raised specifically for meat production, fed feed supplemented with a-lipoic acid and α-tocopherol acetate, and the antioxidant activity and component changes in the meat and meat products were investigated to assess the role of antioxidant supplements on the storage stability of broiler breast meat and meat products.

## Materials and methods

### Chickens, diet and management

One hundred and eighty broiler birds (1 day old), of Hubbard strain, were purchased from the local market. The birds in each experimental unit were kept in separate pens (4 × 3 × 1.5 ft), and the pens were disinfected by using fumigation method. The temperature of the experimental room was maintained at 95 °F during the first week. It was then lowered by 5 °F until it reached 75 °F at the completion of the study. The birds were reared up to 6 weeks. For the first 2 weeks, the birds were fed with the control diet and after that feed was supplemented with ATA (200 mg/kg of feed) and ALA as indicated in treatment plan. The composition of the diet fed to the broilers is depicted in Table 1.

**Table 1:** Feed composition of the diet

**S. No**	**Ingredients and composition of feed**	**Percentage**
1	Corn	30.0
2	Corn germ meal	18.4
3	Soybean meal, 44% protein	17.4
4	Sorghum	10.0
5	Sunflower meal	8.0
6	Barley	5.8
7	Rye	5.0
8	Beef tallow	0.1
9	Calcium carbonate	1.9
10	Dicalcium phosphate	1.4
11	Sepiolite	1.0
12	Salt	0.3
13	Sodium bicarbonate	0.1
	**Composition**	
1	Dry matter	88.2
2	Crude protein	16.0
3	Crude fat	4.1
4	Crude fiber	5.1
5	Ash	7.4

### Slaughtering and sample collection

At the end of the 6 weeks, the birds were slaughteed and sampling of red and white meat was done. Five birds from each feeding group were collected and slaughtered at the farm by cervical dislocation and then the meat was stored at −4 °C in a refrigerator (Sanyo, Japan) for further analysis.

### Sample preparation

Five gram meat sample was taken in 50 ml polypropylene tube having a cap and sample homogenized by using phosphate buffer and glycerol (20%) pH (7.4) with the help of homogenizer. The homogenized breast meat sample were centrifuged at 1000 × g for ten minutes to remove the nuclear fraction after that supernatant was collected in a separate tube and then it was used for further analysis.

### Analysis of breast muscles of broiler meat

The antioxidant activity of breast meat was assessed by measuring their scavenging abilities to DPPH stable radicals as described by the method of [13]. The oxidative stability of breast meat was assessed by measuring mg of malondialdehyde per kg meat. The lipid oxidation was determined as the TBARS value by using the method described by [14]. ALA content of breast was determined by HPLC (gradient) system according to the method described by [15]. ATA of broiler breast meat was determined by HPLC (isocratic) system according to the method described by [16].

### Product development

ALA and ATA enriched broiler breast meat was utilized in the preparation of nuggets and patties.

### Preparation of nuggets

Nuggets were prepared by the method described by [17]. The basic recipe of the nuggets is as (Chicken boneless 500 g, Egg 1, oil As required for frying, Black pepper 12 g, Garlic paste 10 g, Onion 100 g, Plain flour 120 g, Bread crumbs 70 g, salt 20 g).

The raw material in the manufacturing of the nuggets was weighed and cleaned according to the recipe. Fresh and antioxidant enriched broiler breast meat was washed with tap water, deboned manually and was minced by using electric mincer to obtain highly minced meat for the preparation of high textured broiler meat nuggets. First of all broiler meat and onion were mixed in a meat mixer for five minutes, then all other ingredients were added according to the recipe, then all these ingredients were mixed by using meat mixer to provide a uniform blend. When all components were thoroughly mixed then the mixture was extended in a thin layer (10 mm thickness) and shaped into discs of 30 mm diameter (10 ± 1 g/piece) in the Meat Technology Laboratory (National Institute of Food Science and Technology, University of Agriculture, Faisalabad). When nuggets were prepared, these were dipped in plain flour and bread crumbs separately and frying of the nuggets was done in canola oil at 180 °C till golden brown color was appeared.

### Burger patties preparation

The burger Patties were made by the method as described by [18]. The basic recipe of burger patties is as (Boneless chicken 625 g, Fresh breadcrumbs 90 g, Onions 250 g, Tomatoes 150 g, Chopped fresh coriander 45 g, Ginger grated 2.5 g, Clove 1 g, Ground cumin 15 g, Garam masala 15 g, Egg 1, Salt 50 g, Vegetable oil 250 g). The raw material in the manufacturing of the burger patties were weighed and cleaned according to the recipe. Antioxidant enriched broiler breast meat was washed with tap water, deboned manually and was minced by using electric mincer to obtain highly minced meat for the preparation of the good textured broiler meat burger patties. Minced chicken meat and onion were boiled in water for 15 minutes in pressure cooker. First of all minced broiler meat and onion were mixed in a meat mixer for five minutes, then red chilies, spices and salt were added according to the recipe, then all these ingredients were mixed by using meat mixer. After well mixing of all the ingredients, burger patties were made by using burger patty maker of 50 g size, in the Meat Technology lab. When patties were ready for frying, these were rested for some time, followed by frying in canola oil till golden brown color achived.

### Storage of nuggets and patties

ALA and ATA enriched nuggets and patties were vaccum sealed in plastic bags and then stored at −18 °C in a freezer for a storage period of 90 days.

### Analysis of nuggets and burger patties

#### Lipid peroxidation stability by thiobarbituric acid reactive substances (TBARS) assay

The oxidative stability of the breast meat nuggets and burger patties was assessed by measuring mg of malondialdehyde per kg of meat. Five gram sample of nuggets and burger patties was taken in test tube with 15 mL distilled water was homogenized at 1130 × g for 1 minute. Sample homogenate (1 mL) was transferred to a test tube. After that, 50 μL butylated hydroxyanisol (7.2%) and 2 mL TBARS–TCA solution (20 mM TBARS in 15% TCA) were added to the test tube. Tubes were heated (90 °C) in a boiling water bath for 30 min, cooled, and then centrifuged at 2090 × g for 15 min. Absorbance of the supernatant was measured at 532 nm with a spectrophotometer. The lipid oxidation was determined as the TBARS value by using the method described by [14].

### 1, 1-diphenyl-2-picrylhdrazyl (DPPH) free radical scavenging assay

The antioxidant activity of the breast meat nuggets and burger patties was assessed by measuring their scavenging abilities to DPPH stable radicals. A sample of 125 μL was mixed with 0.0012 m DPPH solution followed by the addition of 95% MeOH up to a final volume of 4 mL. The absorbances of the resulting solution and the blank were recorded after 1 h at room temperature. The conversion the color of DPPH was read spectrophotometrically at 515 nm. Inhibition of free radicals by DPPH in percent (%) was calculated in according to the method of [13].

(1) $$\text{I}\left(\%\right)=100\times \left({\text{A}}_{{\text{blank}}_{-}}{\text{A}}_{\text{sample}}/{\text{A}}_{\text{blank}}\right)$$

### Physico chemical analysis of nuggets and patties

The pH of Nuggets and Patties was measured by using pH meter by the method described by [19]. Ten gram of sample was homogenized with 50 mL distilled water and pH value was measured by a digital pH-meter. A hand held tristimulus colorimeter (Color Test Meter II) was used to determine the color of breast Nuggets and Patties at regular storage intervals (0, 10, 20 and 30 days) as described by [20]. Calibration of the colorimeter was done by using standards (54 CTn for dark and 151 °C Tn for light). Color was determined by placing nuggets and patties in petriplate under the photocell. The water activity of breast Nuggets and Patties was determined by using an electronic Hygropalm water activity meter (Model Aw-Win, Rotronic, equipped with a Karl-Fast probe) at regular storage intervals as described by [21]. The sample was taken in plastic cup and the water activity is measured by water activity meter. The textural characteristics of nuggets and patties were analyzed at different storage intervals by means of texture analyzer (Mod. TA-XT2, Stable Microsystems, surrey, UK). Texture was determined with the help of texture analyzer (model TA_XT Plus, Stable Microsystems, Surrey, UK) as described by [22]. The nuggets and patties were fried and compression test was performed to check the texture of the product.

### Sensory evaluation of nuggets and patties

The sensory evaluation of fried nuggets and patties was carried out for the different attributes like Appearance, Flavor, Taste and overall acceptability by using nine point hedonic scale after 0, 30, 60 and 90 days interval, by trained panelists as the method described by [23].

### Statistical analysis

The data obtained for each parameter was subjected to statistical analysis to determine the level of significance according to the method described by [24] by using the software package (Statistic 8.1). The Duncan^’^s multiple range (DMR) test was used to estimate the level of significance that existed between the mean values.

## Results and discussion

### Broiler growth performance

The result depicted in Table 2,3 revealed a highly significant effect of AlA and ATA on weight gain of broilers among all the treatments. Birds in (T_2_) containing minimum dose of alpha-lipoic acid 25 mg gained more weight 1948.25 g whereas T4 containing maximum dose of alpha lipoic acid (150 mg of alpha lipoic acid) gained minimum weight i.e. 1691.25 gm at the end of 42 day trial. Weight gain of the treatment (T_5_) was 1783.63 gm which contained oxidized oil in the feed. The feed conversion ratio (FCR) of the treatment T_2_ was 1.92 and the treatment T_4_ was 2.07. It means that the group containing only oxidized oil had adverse effect on the performance of broiler chicks and the group containing Maximum alpha lipoic acid also reduces the overall weight of the broiler birds. The results of this study are in correlation [25,26] who also observed significantly lower weight gain of broilers fed on diets containing oxidized oil whereas; antioxidant supplementation feed improved the growth of birds.

**Table 2:** Weight gain of the broiler birds fed with alpha lipoic acid and alpha tocopherol acetate enriched feed on weekly basis in (g/week)

**Weeks**	**W 1**	**W 2**	**W 3**	**W 4**	**W 5**	**W 6**
T1	148 ± 1.37	447 ± 2.94	678 ± 3.71	1013 ± 5.98	1385 ± 7.67	1831 ± 16.00
T2	150 ± 1.50	369 ± 2.40	685 ± 9.36	1004 ± 4.52	1378 ± 7.14	1948 ± 6.14
T3	151 ± 1.74	374 ± 1.91	671 ± 2.40	1006 ± 6.01	1392 ± 4.20	1912 ± 11.01
T4	147 ± 0.85	360 ± 3.96	694 ± 3.28	929 ± 5.46	1291 ± 8.70	1691 ± 7.45
T5	147 ± 1.41	345 ± 3.0	675 ± 4.63	984 ± 3.36	1329 ± 5.96	1783 ± 12.61
T6	148 ± 1.11	349 ± 3.60	669 ± 4.42	948 ± 3.06	1315 ± 7.98	1895 ± 4.00
Means	149^F^	374^E^	679.0^D^	981^C^	1348^B^	1843^A^

**Table 3:** Feed Conversion Ratio (FCR) of the experimental broiler birds fed with alpha lipoic acid and alpha tocopherol acetate enriched feed

**Weeks**	**W1**	**W2**	**W3**	**W4**	**W5**	**W6**	**Means**
T_1_	1.61 ± 0.09	1.20 ± 0.01	2.94 ± 0.06	2.01 ± 0.05	2.58 ± 0.02	2.32 ± 0.09	2.10^C^
T_2_	1.61 ± 0.01	1.64 ± 0.02	2.17 ± 0.03	2.04 ± 0.05	2.22 ± 0.02	1.87 ± 0.04	1.92^E^
T_3_	1.60 ± 0.04	1.62 ± 0.03	2.26 ± 0.01	1.92 ± 0.04	2.33 ± 0.04	2.05 ± 0.04	1.96^D^
T_4_	1.99 ± 0.02	2.00 ± 0.03	2.08 ± 0.04	2.71 ± 0.05	2.38 ± 0.09	2.78 ± 0.08	2.32^A^
T_5_	1.80 ± 0.01	1.69 ± 0.05	2.05 ± 0.03	2.09 ± 0.06	2.25 ± 0.03	2.59 ± 0.08	2.07^C^
T_6_	2.12 ± 0.00	2.13 ± 0.04	2.09 ± 0.03	2.38 ± 0.04	2.53 ± 0.05	1.74 ± 0.04	2.16^B^
Means	1.78^E^	1.71^F^	2.26^B^	2.19^D^	2.38^A^	2.22^C^	

### Results of fresh breast meat

The results of DPPH free scavenging assay of breast meat showed that there is significant effect of ALA and ATA on all the treatments. Free radical inhibition percentage of breast meat ranged from 44.98 to 78.69% as showed in Table. Our findings are concurred with the agreement with [27] and [28] they were reported that vitamin E and sage extract increased the antiradical power than control treatment respectively.

Lipid peroxidation is the measurement of MDA compound formed during autoxidation of lipids present in meat. Higher MDA compounds revealed higher amount of lipids, which deteriorate the quality the meat. T4 containing (200 mg ATA and 150 mg ALA) exhibited less MDA (0.1467 ± 0.05^F^) as compared with T_1_(0.1980 ± 0.03^C^), and oxidized oil showed highest MDA(0.2410 ± 0.10^A^) formation in broiler breast meat. Treatments with antioxidants supplemented, ALA and ATA, depicted less peroxidation by producing lower amounts of MDA. Results are agreed with [16,25,29].

Alpha-tocopherol is one of the major lipid soluble antioxidant of tocopherol family which cannot be synthesized by the animal cells [30]. Alpha-tocopherol is a biological antioxidant which protects the membrane from oxidation. The supplementation of alpha-tocopherol in feed of animal increase the deposition as the concentration increase [31]. Alpha-tocopherol acetate content was increased as the supplementation of α-lipoic increased with constant level of α-tocopherol and it ranged from 79.09 to 206.43 mg/g in breast meat on dry basis. The results of this study are in agreement with [5,32] who also stated that the alpha-lipoic acid concentration increase the alpha-tocopherol deposition.

Alpha-lipoic acid (ALA) is a free radical defender and has excellent bioavailability. It is both fat- and water-soluble [33]. Many studies reported that administration of lipoic acid decrease oxidative stress and restore reduce levels of other antioxidants in vivo. It has been reported that as a food supplement Alpha-lipoic acid could have diverse biological functions [34]. The result showed that maximum alpha-lipoic acid deposited in T4 (96.54 mg/mg) in case of leg meat which is revealed from Table 4.17. It also deposits in control (22.69 mg/gram) and oxidized group (17.19 mg/gram) but deposition rate was higher in groups that were fed with higher level of alpha-lipoic acid with constant level of alpha-tocopherol respectively. The alpha-lipoic acid was deposited in the ranged 8.73 to 154.16 mg/gram of breast meat. These results depicted that α-lipoic acid have positive interaction with α-tocopherol, which is agreement with [5,32].

**Table 4:** TBARS Value, DPPH Assay, Alpha lipoic acid content and Alpha tocopherol acetate content in breast meat of broiler birds fed on supplemented diet

**Treatment**	**TBARS values nmol of malondialdehyde/kg of meat**	**DPPH Assay Free radical scavenging activity (%)**	**ΑLA content mg/gram of meat**	**ATA content mg/gram of meat**
T_1_	0.192 ± 0.04^C^	51.3 ± 0.07^D^	29.3 ± 0.04	72.5 ± 0.05^F^
T_2_	0.174 ± 0.03^D^	62.9 ± 0.09^C^	37.1 ± 0.06^C^	89.4 ± 0.08^C^
T_3_	0.161 ±0.02^E^	71.5 ± 0.12^B^	57.1 ± 0.07^B^	102 ± 0.11^B^
T_4_	0.146 ± 0.01^F^	76.7 ± 0.14^A^	154 ± 0.32^A^	206 ± 0.22^A^
T_5_	0.248 ± 0.07^A^	45. ± 0.0.04^E^	8.73 ± 0.02^D^	79.1 ± 0.06^E^
T_6_	0.225 ± 0.05^B^	47.7 ± 0.05^D^	37.4 ± 0.06^C^	84.9 ± 0.07^D^

Data are means of three replicates ± Standard Deviation. Means in the same row have the same letter are not significantly different at 0.05.

### Results of meat products

#### Oxidative stability of nuggets and patties

Lipid peroxidation is one of the major mechanisms of quality deterioration in foods. The changes in quality attribute results deterioration in flavor, color, texture, and nutritive value of the food due to the production of toxic compounds [1]. Cooking process and salt addition enhances lipid peroxidation and ultimately less stability enhancement is observed in nuggets and patties as compared to fresh meat.

### Effect on TBARS and DPPH value of nuggets and burger patties

The results in Table 5 showed that a highly significant effect on TBARS value was observed in the breast chicken nuggets and patties after ALA and ATA incorporation through feed. Chicken nuggets containing ALA and ATA (antioxidant) maintained lower TBARS values throughout the storage period. Lowest TBARS values of breast nuggets were observed in T_4_ (0.18 ± 0.02) which contain maximum amount of alpha lipoic acid and highest TBARS values were observed in T_5_ (0.36 ± 0.05) at 0 day which contain only oxidized oil. The same pattern was followed by the breast meat patties. The lowest TBARS values in T_4_ were due to the antioxidants addition of ALA and ATA. Our findings are concurred with the agreement with [27] and [28] they were reported that vitamin E and sage extract increased the antiradical power than control treatment respectively. A highly significant effect on DPPH assay was observed in the chicken nuggets and patties after ALA and ATA incorporation through feed. Chicken nuggets containing ALA and ATA (antioxidants) maintained higher DPPH percentage inhibition throughout the storage period. The DPPH percentage inhibition values of the breast nuggets were higher as compared to leg nuggets because it contains less amount of fat. Highest DPPH percentage inhibition values of breast nuggets were observed in T_4_ (60.34^A^) which contain maximum amount of alpha lipoic acid and highest and lowest DPPH percentage inhibition values were observed in T_5_ (40.48^E^) at 0 day which contain oxidized oil. The percentage inhibition of breast meat ranged from (44.81 ± 1.02) in T_5_ to (64.67 ± 3.57) in T_4_. The production of free radicals was also lowest at the start of the storage and increase with the passage of the time. Our findings are also very good agreement with the results reported by [27,28] they were reported that vitamin E and sage extract increased the antiradical power than control treatment respectively.

**Table 5:** Antioxidant potential of breast nuggets and patties performed by TBARS and DPPH assay method

**Treatment**	**Storage days**									
**TBARSRS values**	**Broiler meat Nuggets**					**Broiler meat Patties**				
	**0 day**	**30 day**	**60 day**	**90 day**	**Mean**	**0 day**	**30 day**	**60 day**	**90 day**	**Mean**
T_1_	0.202 ± 0.01	0.30 ± 0.02	0.33 ± 0.04	0.39 ± 0.02	0.30BC	0.20 ± 0.01	0.30 ± 0.02	0.33 ± 0.04	0.39 ± 0.02	0.30B
T_2_	0.161 ± 0.02	0.25 ± 0.02	0.31 ± 0.01	0.36 ± 0.01	0.27C	0.16 ± 0.02	0.25 ± 0.02	0.31 ± 0.01	0.36 ± 0.01	0.27C
T_3_	0.17 ± 0.01	0.24 ± 0.03	0.27 ± 0.02	0.35 ± 0.03	0.25C	0.17 ± 0.01	0.24 ± 0.03	0.27 ± 0.02	0.35 ± 0.02	0.25C
T_4_	0.18 ± 0.02	0.21 ± 0.02	0.23 ± 0.03	0.25 ± 0.01	0.46AB	0.18 ± 0.02	0.21 ± 0.02	0.23 ± 0.03	0.25 ± 0.01	0.21D
T_5_	0.36 ± 0.05	0.51 ± 0.02	0.54 ± 0.06	0.65 ± 0.05	0.51A	0.36 ± 0.05	0.50 ± 0.01	0.53 ± 0.05	0.66 ± 0.06	0.51A
T_6_	0.22 ± 0.02	0.27 ± 0.03	0.32 ± 0.03	0.40 ± 0.02	0.30BC	0.22 ± 0.03	0.26 ± 0.03	0.32 ± 0.03	0.40 ± 0.02	0.30B
Mean	0.37A	0.29A	0.33A	0.40A		0.21D	0.29C	0.33B	0.40A	
DPPH Assay										
T_1_	54.23 ± 3.37	52.81 ± 3.35	49.51 ± 3.35	45.11 ± 0.98	50.41^C^	54.09 ± 2.48	53.14 ± 2.48	51.73 ± 2.48	49.84 ± 2.65	52.20^B^
T_2_	51.89 ± 1.23	50.47 ± 1.16	47.17 ± 1.16	40.72 ± 1.35	47.56^D^	51.89 ± 1.16	50.94 ± 1.16	49.53 ± 1.36	47.64 ± 1.18	50.00^B^
T_3_	62.11 ± 0.65	60.69 ± 0.97	57.39 ± 0.97	50.94 ± 1.39	57.78^B^	62.11 ± 0.97	61.16 ± 0.47	59.75 ± 0.97	57.86 ± 0.98	60.22_A_
T_4_	64.67 ± 4.00	63.25 ± 3.57	59.95 ± 3.57	53.51 ± 5.56	60.34^A^	64.67 ± 3.57	63.73 ± 3.57	62.31 ± 3.57	59.83 ± 3.61	62.63^A^
T_5_	44.81 ± 1.05	43.40 ± 1.02	40.09 ± 1.02	33.65 ± 1.74	40.48^E^	44.81 ± 1.02	43.87 ± 1.02	42.45 ± 1.02	45.91 ± 8.65	44.26^C^
T_6_	53.93 ± 1.43	52.52 ± 1.56	49.21 ± 1.56	41.98 ± 1.39	49.41^CD^	53.93 ± 1.56	52.99 ± 1.56	51.57 ± 1.56	51.42 ± 3.79	52.47^B^
Mean	55.27 ± 1.98A	53.85^A^	50.55^B^	44.31^C^		55.25^A^	54.30^AB^	52.89^B^	52.08^B^	

Data are means of three replicates ± Standard Deviation. Means in the same row have the same letter are not significantly different at 0.05.

### Physico chemical characteristics of nuggets and patties

#### pH and water activity

The Table 6 showed that there is a highly significant effect of ALA and ATA treatments on the pH of broiler meat nuggets and patties. The effect of days on the pH of broiler meat products was also highly significant. It is obvious from results that pH value of breast nuggets varied from 5.98-6.78 due to the fact variation in treatments. The pH range of breast patties was that of 5.85-6.72. The pH values of the nuggets and patties increases with passages of the time. The increase in pH value during storage period revealed that, there was significant breakdown of meat protein on storage of the product. Our findings also match with [35,36] who also observed increase in pH with the passage of the time in the nuggets containing antioxidants.

**Table 6:** pH and water activity of breast nuggets and patties at various sorage intervals stored at −18 °C

**Treatment**	**Storage days**									
**pH**	**Broiler meat Nuggets**					**Broiler meat Patties**				
	**0 day**	**30 day**	**60 day**	**90 day**	**Mean**	**0 day**	**30 day**	**60 day**	**90 day**	**Mean**
T_1_	6.14 ± 0.03	6.20 ± 0.01	6.25 ± 0.02	6.30 ± 0.03	6.22^D^	6.07 ± 0.07	6.03 ± 0.05	6.11 ± 0.04	6.22 ± 0.03	6.10^C^
T_2_	5.92 ± 0.04	5.95 ± 0.01	6.02 ± 0.02	6.08 ± 0.03	5.99^E^	5.82 ± 0.05	5.89 ± 0.09	5.93 ± 0.05	6.01 ± 0.10	5.91^E^
T_3_	5.99 ± 0.11	5.95 ± 0.02	5.98 ± 0.01	6.04 ± 0.03	5.98^E^	5.92 ± 0.05	6.00 ± 0.10	6.07 ± 0.09	6.12 ± 0.06	6.02^D^
T_4_	6.19 ± 0.05	6.27 ± 0.02	6.36 ± 0.01	6.46 ± 0.03	6.31^B^	6.24 ± 0.03	6.30 ± 0.06	6.38 ± 0.07	6.39 ± 0.05	6.32^B^
T_5_	6.38 ± 0.02	6.41 ± 0.01	6.54 ± 0.02	6.72 ± 0.04	6.51^A^	6.43 ± 0.07	6.55 ± 0.09	6.65 ± 0.04	6.72 ± 0.03	6.58^A^
T_6_	6.20 ± 0.02	6.22 ± 0.01	6.30 ± 0.02	6.31 ± 0.06	6.25^C^	6.00 ± 0.10	6.22 ± 0.25	6.19 ± 0.04	6.28 ± 0.03	6.17^C^
Mean	6.13^D^	6.16^C^	6.24^B^	6.31^A^		6.08^D^	6.16^C^	6.22^B^	6.28^A^	6.10^C^
Water Activity										
T_1_	0.81 ± 0.01	0.79 ± 0.01	0.75 ± 0.02	0.72 ± 0.01	0.76^CD^	0.81 ± 0.01	0.79 ± 0.01	0.75 ± 0.02	0.72 ± 0.01	0.76^A^
T_2_	0.83 ± 0.01	0.80 ± 0.01	0.74 ± 0.02	0.72 ± 0.03	0.77^BC^	0.83 ± 0.01	0.80 ± 0.01	0.74 ± 0.02	0.72 ± 0.01	0.77^B^
T_3_	0.84 ± 0.01	0.81 ± 0.01	0.74 ± 0.02	0.73 ± 0.01	0.78^B^	0.84 ± 0.01	0.81 ± 0.01	0.74 ± 0.02	0.73 ± 0.01	0.78^B^
T_4_	0.85 ± 0.02	0.82 ± 0.01	0.78 ± 0.01	0.75 ± 0.01	0.79^A^	0.85 ± 0.02	0.82 ± 0.01	0.78 ± 0.01	0.75 ± 0.01	0.79^B^
T_5_	0.81 ± 0.01	0.76 ± 0.01	0.70 ± 0.01	0.70 ± 0.02	0.74^E^	0.81 ± 0.01	0.76 ± 0.01	0.70 ± 0.01	0.70 ± 0.02	0.74^B^
T_6_	0.83 ± 0.01	0.78 ± 0.02	0.74 ± 0.01	0.70 ± 0.01	0.76^D^	0.83 ± 0.01	0.78 ± 0.02	0.74 ± 0.01	0.70 ± 0.01	0.76^B^
Mean	0.82^A^	0.79^B^	0.74^C^	0.72^D^		0.99^A^	0.79^B^	0.74^B^	0.72^B^	

Data are means of three replicates ± Standard Deviation. Means in the same row have the same letter are not significantly different at 0.05.

The result showed that the different treatments of alpha lipoic acid, alpha tocopherol acetate and oxidized oil have highly significant effect on the water activity of antioxidant enriched boiler meat nuggets and patties. Water of breast nuggets in T_4_ (0.85 ± 0.02) and inT_5_ was (0.81 ± 0.01) at 0 day storage interval. The water activity of the nuggets and patties decreases and product move towards hardness with the passage of the time. This result correlate with the result of [37] who studied that water activity of crusted dry-cured loin and non-crusted dry-cured loin decrease and the texture of the product go towards hardness with advancement of the storage period.

### Color and texture

The Table 7 of broiler breast meat nuggets and patties of color showed that there is a highly significant effect of ALA and ATA treatments on the color of breast meat nuggets and patties. The color of the patties and nuggets was light at the start of the storage period and with the passage of the time the color of the meat nuggets and patties become dark. The color of breast meat nuggets ranged from 94.33-115and the color of breast meat patties ranged from 96.67-115.33. During storage the values for the color decreased with the passage of time. The decrease in value indicated that the nuggets and patties become darker during storage. Our results are in agree with [38] who stated that color value of the chicken patties decrease with passage of the time and color of the patties change from red to brown which could be due to the formation of metmyoglobin in salt containing treatments. The analysis of variance showed that treatments have significant effect on texture properties of nuggets and patties at freezing conditions. However the storage days and their interactive effect of treatments and storage days have significant effect on the texture properties of nuggets and patties. The results of present study are in close collaboration with [37] who stated that the texture value varied significantly with storage days.

**Table 7:** **Color and texture of breast nuggets and patties stored at −18** °C **for a storage period of 90 days**

**Treatment**	**Storage days**									
**Color**	**Broiler meat nuggets**					**Broiler meat patties**				
	**0 day**	**30 day**	**60 day**	**90 day**	**Mean**	**0 day**	**30 day**	**60 day**	**90 day**	**Mean**
**T**_**1**_	112 ± 1.15	109 ± 1.53	105 ± 1.53	100 ± 1.52	106.92^B^	112 ± 0.58	108 ± 0.58	106 ± 1.53	101 ± 1.73	107.08^C^
**T**_**2**_	113 ± 2.00	110 ± 2.00	106 ± 2.02	101 ± 1.99	107.50^B^	113 ± 0.58	110 ± 0.58	107 ± 1.15	104 ± 2.08	109.00^B^
**T**_**3**_	115 ± 1.00	110 ± 1.53	106 ± 1.56	101 ± 1.54	108.50^B^	115 ± 0.58	111 ± 0.58	108 ± 0.58	103 ± 0.58	109.67^B^
**T**_**4**_	116 ± 1.73	112 ± 2.52	108 ± 2.52	103 ± 2.53	110.00^A^	116 ± 0.58	112 ± 0.58	109 ± 1.00	105 ± 0.58	110.92^A^
**T**_**5**_	108 ± 2.00	103 ± 3.51	99 ± 3.51	94 ± 3.50	101.25^C^	106 ± 2.08	101 ± 1.53	99 ± 0.58	96 ± 0.58	101.00^E^
**T**_**6**_	109 ± 1.53	104 ± 3.06	100 ± 3.01	95 ± 3.04	102.42^C^	108 ± 1.54	102 ± 0.58	100 ± 0.58	97 ± 1.15	102.17^D^
**Mean**	112.39^A^	108.33^B^	104.33^C^	99.33^D^	106.92^B^	112.11^A^	107.83^B^	105.17^C^	101.44^D^	107.08^C^
**Texture**										
**T**_**1**_	1110 ± 2.5	1118 ± 2.5	1125 ± 2.52	1134 ± 2.52	1122.1^C^	1143 ± 2.52	1149 ± 2.63	1158 ± 3.21	1165 ± 2.43	1147.8^C^
**T**_**2**_	1120 ± 4.0	1128 ± 4.0	1135 ± 4.10	1144 ± 4.00	1131.8^A^	1153 ± 4.00	1159 ± 3.23	1168 ± 3.52	1175 ± 4.12	1157.5^A^
**T**_**3**_	1115 ± 2.1	1123 ± 2.1	1130 ± 2.08	1139 ± 2.08	1127.1^B^	1148 ± 2.08	1154 ± 2.45	1163 ± 2.65	1170 ± 3.67	1152.8^B^
**T**_**4**_	1113 ± 3.1	1121 ± 3.1	1128 ± 3.06	1137 ± 3.06	1125.1^B^	1146 ± 3.06	1152 ± 3.87	1161 ± 3.98	1168 ± 1.32	1150.8^B^
**T**_**5**_	1108 ± 1.0	1116 ± 1.1	1123 ± 1.09	1132 ± 1.00	1119.8^D^	1141 ± 1.00	1147 ± 1.54	1156 ± 1.63	1163 ± 1.32	1145.5^D^
**T**_**6**_	1115 ± 1.1	1123 ± 1.1	1130 ± 1.00	1139 ± 1.00	1126.8^B^	1148 ± 1.00	1154 ± 1.43	1163 ± 1.47	1170 ± 1.23	1152.5^B^
**Mean**	1113.7^D^	1121.7^C^	1128.7^B^	1137.7^A^		1138.7^D^	1147.7^C^	1156.7^B^	1161.7^A^	

Data are means of three replicates ± Standard Deviation. Means in the same row have the same letter are not significantly different at 0.05.

### Effect on sensory parameters of nuggets

Sensory evaluation of food has been defined as a scientific method used to evoke, measure, analyze and interpret responses to products as perceived through the senses of sight, touch, smell, taste, and hearing. Table 8 showed that there is a highly significant effect of ALA and ATA treatments on all the sensory parameters (appearance, flavor, Taste and overall acceptability) of broiler meat nuggets and patties. Breast chicken nuggets and patties containing ALA and ATA scored significantly (P < 0.05) higher for appearance, flavor, taste and overall acceptability than all other preparation. T_5_ nuggets and patties got least score by sensory evaluation because of the rancid taste imparted by the oxidized oil. The appearance of the nuggets and patties decreases with passage of the time. The minimum value for flavor was observed in T_5_ (5.25) at day 30 because it contain oxidized oil and it impart a rancid flavor to the products. The decrease in flavor value is due to the peroxidation of PUFA which results in rancid odors and flavors. Decrease in flavor scores might be due to development of oxidative rancidity and microbial deterioration in products during storage. During storage the score for taste of nuggets and patties decreased because with the passage of time lipid peroxidaion of the nuggets and patties was enhanced and it decreases the taste of the products. The decreasing trend in taste of nuggets may be associated with the peroxidation of PUFA. The results of the study correlate with [36,39] who also reported that the taste of the nuggets decreased significantly with the advancement of storage period. All sensory scores decreased significantly (P < 0.05) with the advancement of storage period. The results of the mean tables of the nuggets and patties showed that T4 was highly acceptable by the panelists as it got highest score for overall acceptability while T5 got minimum score for the overall acceptability. My results are in correlation with 36, 39) also reported that all the sensory quality values decreased significantly with the advancement of storage period.

**Table 8:** Appearance, Flavor, Taste and overall acceptability of breast nuggets and patties stored at −18 °C

**Treatment**	**Storage days**									
**Appearance**	**Broiler meat nuggets**					**Broiler meat patties**				
	**0 day**	**30 day**	**60 day**	**90 day**	**Mean**	**0 day**	**30 day**	**60 day**	**90 day**	**Mean**
T_1_	8.2 ± 0.36	7.8 ± 0.33	7.5 ± 0.43	7.2 ± 0.28	7.1^B^	8.3 ± 0.28	7.8 ± 0.33	7.5 ± 0.43	7.2 ± 0.28	7.1^B^
T_2_	7.7 ± 0.36	7.2 ± 0.13	7.0 ± 0.48	6.7 ± 0.26	6.8^C^	7.7 ± 0.36	7.1 ± 0.14	7.0 ± 0.48	6.9 ± 0.13	6.7^C^
T_3_	6.7 ± 0.33	6.8 ± 0.64	6.0 ± 0.43	5.7 ± 0.26	6.8^C^	6.7 ± 0.33	7.1 ± 0.75	6.5 ± 0.50	6.2 ± 0.67	6.8^C^
T_4_	8.2 ± 0.36	7.8 ± 0.30	7.4 ± 0.29	7.3 ± 0.18	7.6^A^	8.3 ± 0.23	7.8 ± 0.30	7.4 ± 0.29	7.3 ± 0.18	7.5^A^
T_5_	6.2 ± 0.36	5.8 ± 0.33	5.5 ± 0.43	5.2 ± 0.26	6.7^C^	6.2 ± 0.36	5.8 ± 0.33	5.5 ± 0.43	5.2 ± 0.26	6.7^C^
T_6_	7.2 ± 0.34	6.8 ± 0.33	6.5 ± 0.46	6.2 ± 0.19	6.9^C^	7.2 ± 0.34	6.8 ± 0.33	6.5 ± 0.46	6.1 ± 0.33	6.8^C^
Mean	7.4^A^	7.3^A^	6.7^B^	6.5^C^		7.4^A^	7.2^A^	6.7^B^	6.5^B^	7.1^B^
**Flavor**										
T_1_	8.2 ± 0.36	7.8 ± 0.33	7.5 ± 0.43	7.2 ± 0.28	7.6^A^	8.3 ± 0.28	7.8 ± 0.33	7.5 ± 0.43	7.2 ± 0.28	7.20^A^
T_2_	7.7 ± 0.36	7.2 ± 0.13	7.0 ± 0.48	6.7 ± 0.26	7.1^B^	7.7 ± 0.36	7.1 ± 0.14	7.0 ± 0.48	6.9 ± 0.13	6.8^B^
T_3_	6.7 ± 0.33	6.8 ± 0.64	6.0 ± 0.43	5.7 ± 0.26	6.3^D^	6.7 ± 0.33	7.1 ± 0.75	6.5 ± 0.50	6.2 ± 0.67	6.0^C^
T_4_	8.2 ± 0.36	7.8 ± 0.30	7.4 ± 0.29	7.3 ± 0.18	7.6^A^	8.3 ± 0.23	7.8 ± 0.30	7.4 ± 0.29	7.3 ± 0.18	7.2^A^
T_5_	6.2 ± 0.36	5.8 ± 0.33	5.5 ± 0.43	5.2 ± 0.26	5.6^E^	6.2 ± 0.36	5.8 ± 0.33	5.5 ± 0.43	5.2 ± 0.26	5.5^D^
T_6_	7.2 ± 0.34	6.8 ± 0.33	6.5 ± 0.46	6.2 ± 0.19	6.6^C^	7.2 ± 0.34	6.8 ± 0.33	6.5 ± 0.46	6.1 ± 0.33	6.3^C^
Mean	7.4^A^	7.0^B^	6.6^C^	6.4^C^		7.2^A^	6.7^B^	6.4^B^	5.8^C^	7.20^A^
**Taste**										
T_1_	7.2 ± 0.20	6.7 ± 0.16	6.5 ± 0.13	6.3 ± 0.12	6.7^B^	7.2 ± 0.41	6.7 ± 0.16	6.5 ± 0.13	6.3 ± 0.12	6.7^B^
T_2_	6.2 ± 0.30	6.2 ± 0.17	6.0 ± 0.13	5.8 ± 0.16	6.0^C^	6.2 ± 0.20	6.2 ± 0.13	6.0 ± 0.14	5.8 ± 0.16	6.1^C^
T_3_	8.2 ± 0.40	7.7 ± 0.18	7.5 ± 0.16	7.3 ± 0.12	7.6^A^	8.2 ± 0.12	7.7 ± 0.12	7.5 ± 0.10	7.3 ± 0.12	7.7^A^
T_4_	7.7 ± 0.20	6.7 ± 0.16	6.5 ± 0.13	6.2 ± 0.06	6.8^B^	7.7 ± 0.22	6.7 ± 0.16	6.5 ± 0.17	6.2 ± 0.06	6.8^B^
T_5_	5.7 ± 0.50	5.8 ± 0.10	5.6 ± 0.08	5.3 ± 0.12	5.5^D^	5.7 ± 0.25	5.8 ± 0.13	5.6 ± 0.13	5.3 ± 0.12	5.6^D^
T_6_	6.7 ± 0.60	6.2 ± 0.19	6.0 ± 0.13	5.8 ± 0.16	6.2^C^	6.7 ± 0.40	6.2 ± 0.16	6.0 ± 0.19	5.8 ± 0.16	6.2^C^
Mean	6.9^A^	6.6^B^	6.3^C^	6.1^D^	6.7^B^	7.0^A^	6.6^B^	6.3^C^	6.1^D^	
**Overall acceptability**										
T_1_	7.7 ± 0.36	7.2 ± 0.15	7.0 ± 0.14	6.8 ± 0.25	7.2^B^	7.9 ± 0.36	7.2 ± 0.15	7.0 ± 0.14	7.0 ± 0.12	7.2^B^
T_2_	7.2 ± 0.36	6.7 ± 0.22	6.5 ± 0.09	6.3 ± 0.25	6.7^D^	7.4 ± 0.36	6.7 ± 0.22	6.5 ± 0.09	6.4 ± 0.17	6.7^CD^
T_3_	7.7 ± 0.33	6.9 ± 0.22	6.5 ± 0.14	6.4 ± 0.31	6.8^C^	7.7 ± 0.35	6.9 ± 0.22	6.5 ± 0.14	6.4 ± 0.31	6.8^C^
T_4_	8.5 ± 0.20	7.7 ± 0.24	7.5 ± 0.09	7.3 ± 0.19	7.7^A^	8.7 ± 0.36	7.7 ± 0.22	7.5 ± 0.09	7.2 ± 0.10	7.8^A^
T_5_	6.8 ± 0.25	6.2 ± 0.23	6.0 ± 0.17	5.8 ± 0.19	6.2^E^	6.8 ± 0.25	6.3 ± 0.31	6.0 ± 0.17	5.8 ± 0.19	6.2^E^
T_6_	7.2 ± 0.33	6.5 ± 0.22	6.5 ± 0.14	6.3 ± 0.15	6.6^D^	7.2 ± 0.33	6.5 ± 0.27	6.5 ± 0.14	6.3 ± 0.15	6.6^D^
Mean	7.5^A^	6.9^B^	6.6^C^	6.4^D^	Mean	7.6^A^	6.9^B^	6.6^C^	6.5^C^	

Data are means of three replicates ± Standard Deviation. Means in the same row have the same letter are not significantly different at 0.05.

## Conclusion

The results from the present study revealed that the antioxidant potential of the breast meat and meat products of the broilers can be improved by supplementing ALA and ATA through feed supplementation. The 150 mg/kg feed dietary level of ALA and ATA (200 mg/kg feed) supplementation gives best results and improved the antioxidant potential and enhances the oxidative stability of meat and meat products. It is also evident from results that nuggets and patties can be stored at (−18 °C) ± 2 °C for 3 months or more. Future research is necessary in respect that Clinical trial should be conducted in future for the evaluation of the antioxidant enriched broiler meat.

## Abbreviation

MDA: malondialdehydes; ALA: α-lipoic acid; ATA: α –tocopherol acetate; TBARS: thiobarbituric acid reactive oxygen species; HPLC: high-performance liquid chromatography.

## Authors’ contribution

The contribution of the each author for this paper was as follows, MSA, FMAB, MIKC, MSAD, MSE, AD carried out the trial of the broiler birds and also collected all the data of the trial. They also arranged all the data and drafted the manuscript. B acting as principal investigator of the project provides technical assistance during research of the broiler and also guided in the analysis and statistical design of research trial. CE also helped to carry out the analytical research work and analysis of the meat and meat products. “It is also confirmed that all the authors read and approved the final manuscript".
